# Hybrid physics-machine learning models for quantitative electron diffraction refinements

**DOI:** 10.1038/s41467-026-71673-9

**Published:** 2026-04-11

**Authors:** Shreshth A. Malik, Tiarnan A. S. Doherty, Benjamin Colmey, Stephen J. Roberts, Yarin Gal, Paul A. Midgley

**Affiliations:** 1 OATML, Department of Computer Science, University of Oxford, Oxford, UK; 2 Department of Materials Science and Metallurgy, University of Cambridge, Cambridge, UK; 3 Machine Learning Research Group, Department of Engineering Science, University of Oxford, Oxford, UK

**Keywords:** Characterization and analytical techniques, Materials science, Techniques and instrumentation

## Abstract

High accuracy electron microscopy simulations required for quantitative crystal structure refinements face a fundamental challenge: while physical interactions are well-described theoretically, real-world experimental effects are challenging to model analytically. To address this gap, we present a hybrid physics-machine learning framework that integrates differentiable physical simulations with neural networks. By leveraging automatic differentiation throughout the simulation pipeline, our method enables gradient-based joint optimization of physical parameters and neural network components representing experimental variables, offering superior scalability compared to traditional second-order methods. We demonstrate this framework through application to three-dimensional electron diffraction (3D-ED) structure refinement, where our approach learns complex thickness distributions directly from diffraction data rather than relying on simplified geometric models. This method achieves state-of-the-art refinement performance across synthetic and experimental datasets, recovering atomic positions, thermal displacements, and thickness profiles with high fidelity. The modular architecture proposed can naturally be extended to accommodate additional physical phenomena and extended to other electron microscopy techniques. This establishes differentiable hybrid modeling as a powerful paradigm for quantitative electron microscopy, where experimental complexities have historically limited analysis.

## Abstract (web summary)

A hybrid physics-machine learning framework enables scalable dynamical refinement of 3D-ED data by combining differentiable diffraction simulations with neural networks to jointly refine crystal structures and complex experimental parameters.

## Introduction

Recent advances in scientific machine learning have demonstrated remarkable potential to complement traditional physics-based simulations across diverse domains of computational science. Hybrid physics-machine learning (ML) approaches, which combine quantitative physical models with the expressive capacity of neural networks^1–5^, offer a principled framework for addressing complex phenomena that remain challenging for purely analytical methods.

In these approaches, well-established physical theories that govern the forward model of a simulation are explicitly used to maintain theoretical rigor and interpretability, while neural networks are used as universal function approximators^6^ to parameterize complex, system-specific effects that are difficult to model explicitly. Thus these hybrid methods bridge the gap between first-principles theory and experimental reality. In recent years, this approach has shown substantial promise in fields ranging from dynamical systems^7–9^ and atmospheric physics^10^ to quantum physics^11,12^, where the learned components augment traditional simulators to improve realism, accuracy, and/or computational efficiency.

A key enabler of these innovations is differentiable physics, in which physical simulators are constructed to support gradient evaluation through every computational step. In traditional physics-based parameter refinement, the application of gradient-based optimization requires explicitly deriving and implementing analytical expressions for the derivatives of the forward model. This process naturally becomes prohibitively complex for large-scale systems and more complex forward model theories. Automatic differentiation^13–16^ overcomes this limitation by systematically applying the chain rule to the elementary operations comprising the simulation to ‘backpropagate’ gradients to the parameters of interest with a computational cost comparable to that of the forward model, without the need for analytic derivative computation. Thus by implementing the forward model within modern automatic differentiation frameworks such as PyTorch^17^ or JAX^18^, the backpropagated gradients can be used to efficiently refine both physical parameters and neural network components jointly within a unified, end-to-end optimization framework.

Electron microscopy represents an ideal setting for the use of a hybrid modeling approach because while the elastic interaction of the electron beam with matter is well described by established physical theory^19,20^, real experiments involve sample morphologies, inelastic scattering, electron beam damage, atomic scale defects and other sample heterogeneities that are notoriously difficult to parameterize. Omitting these contributions in simulations imposes a systematic ceiling on the precision and accuracy with which structural and compositional information can be recovered from experimental data. However, to the best of our knowledge, there has not yet been a demonstration of the utility of hybrid modeling approaches when applied to any electron microscopy technique.

**Fig. 1:**
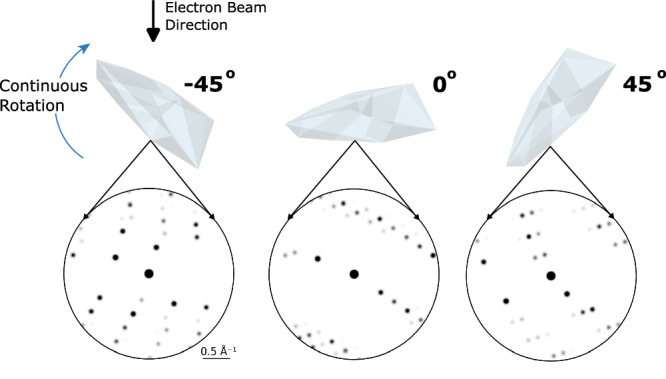
Schematic of the 3D-ED method. An irregularly shaped crystal is continuously rotated under the electron beam while diffraction patterns are recorded. Three representative orientations of a quartz crystal with corresponding simulated dynamical diffraction patterns are shown.

One such electron microscopy modality that could benefit substantially from a hybrid modeling approach is the field of three dimensional electron diffraction (3D-ED), where a crystal is rotated relative to an electron beam and a diffraction pattern is obtained at every orientation (Fig. 1). 3D-ED has emerged as a compelling quantitative technique for structure determination in molecular and materials science^21^, particularly for nanocrystalline samples unsuitable for traditional X-ray analysis. The interaction of electrons with matter, which is Coulombic in nature, is orders of magnitude stronger than that of X-rays^22^; this allows high-resolution diffraction patterns to be obtained from *μ*m or even nm scale crystals^23,24^ and drives pronounced ‘dynamical’ interactions between the incident and diffracted beams, so that the experimentally recorded intensities become exquisitely sensitive to subtle variations in the underlying structure factors^19,25^. Practically, this extreme sensitivity enables the precise location of weakly scattering hydrogen atoms^21^, robust determination of the absolute structure of chiral systems^26^, unambiguous discrimination between atomic species possessing nearly identical scattering factors^27^ and even information on charge density deformation due to bonding to be obtained^28^, provided that dynamical calculations are performed and incorporated into the refinement process in order to accurately interpret the experimental data.

For dynamical refinements of 3D-ED data, the Bloch wave formalism^19,29^ is typically employed to simulate diffracted intensities from a structural starting model, compare these intensities to those observed experimentally, update the model and repeat the process until convergence is achieved^30^. While the Bloch wave approach provides a physically accurate description of multiple elastic scattering, each evaluation of the scattering matrix scales as ${{{\mathcal{O}}}}({N}^{3})$ and a full 3D-ED refinement, which typically involves 10^3^–10^5^ evaluations, can quickly scale to hours or even days. As dynamical diffracted intensities are highly sensitive to thickness variations^31^, accurate modeling of how thickness varies as a function of sample orientation throughout the dataset is essential for quantitative refinement^28^. Real crystals possess irregular shapes, bending, surface roughness, and defects that induce complex, thickness profiles that vary as a function of crystal orientation.

In this work, we introduce a hybrid physics-machine learning approach that integrates a neural-network that models these complex thickness profiles with a fully differentiable implementation of the Bloch-wave formalism^13,32^. While the Bloch-wave component governs the rigorous calculation of dynamical diffracted intensities given a structural model, the neural network augments this by learning orientation-dependent thickness parameters directly from data. Crucially, the differentiability of the Bloch-wave solver enables gradients of the simulated intensities to be backpropagated automatically through both the scattering calculation and the learned thickness model. This facilitates the use of gradient-based optimization to jointly refine structural parameters, thermal displacements, and thickness variations within a single, end-to-end framework.

We demonstrate that this hybrid differentiable framework matches or exceeds the performance of traditional refinements on both synthetic and experimental 3D ED datasets. By leveraging first-order optimizers, which incur only linear storage and compute costs, we can co refine thousands of thickness model parameters alongside structural  parameters in a single pass. This scalability, which is infeasible with traditional Hessian-based, second order schemes, is a direct benefit of the differentiable formulation. While our focus here is on 3D ED, the same differentiable physics plus learned submodel strategy can be applied to other electron microscopy modalities (e.g., 4D STEM, convergent beam diffraction, scanning precession ED) where complex, hard to model experimental effects likewise limit quantitative analysis.

## Results

### Refinement workflow

**Fig. 2:**
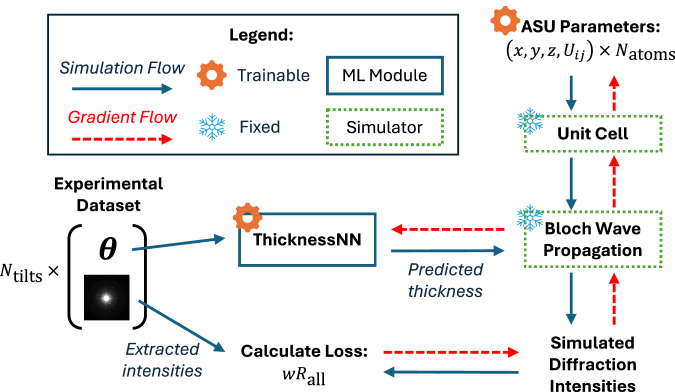
Schematic diagram for the proposed hybrid physics-machine learning model for dynamical electron diffraction. Spatial (*x*, *y*, *z*) and thermal displacement (*U*_*ij*_) parameters of the atoms in the asymmetric unit (ASU), along with neural network parameters of the thickness neural network (ThicknessNN) are jointly optimized via backpropagation to minimize the diffraction loss *wR*_all_.

Figure 2 is a graphical overview of our differentiable refinement workflow. Beginning with the asymmetric unit (ASU), we apply space group symmetry operations to expand the ASU into the unit cell. The unit cell is then rotated to an orientation that matches the experimentally observed diffraction pattern. Following this, we perform a Bloch wave calculation to obtain the simulated intensities at this crystal orientation, where the sample thickness used in the simulation is determined by a lightweight neural network (ThicknessNN, Thickness modelling section). A loss is then computed – in this case the crystallographic R-factor – between the integrated observed and simulated intensities. Using backpropagation, we evaluate the gradients of this loss with respect to the ASU parameters, including atomic positions and isotropic or anisotropic displacement parameters, and the ThicknessNN. Finally, these parameters are updated via gradient descent to minimize the loss.

The refinement is carried out against experimental integrated intensities, obtained after the full reduction of the raw diffraction patterns. This involves indexing and extracting per-hkl intensities with uncertainties informed by detector gain and noise characteristics, and the variance obtained from the background pixels surrounding each Bragg disc^33^. The data are then organised into virtual frames by segmenting the tilt series into angular ranges, and each hkl is allocated to the appropriate frame to allow direct matching of simulated and experimental hkl, with refinements carried out on a frame-by-frame basis, as described in Ref. ^30,33–35^.

All datasets contained in this paper are continuous rotation type datasets, with the exception of the Paracetamol experimental dataset presented in Refinement on experimental data section, which is a precession assisted rotation dataset.

### Thickness modelling

**Fig. 3:**
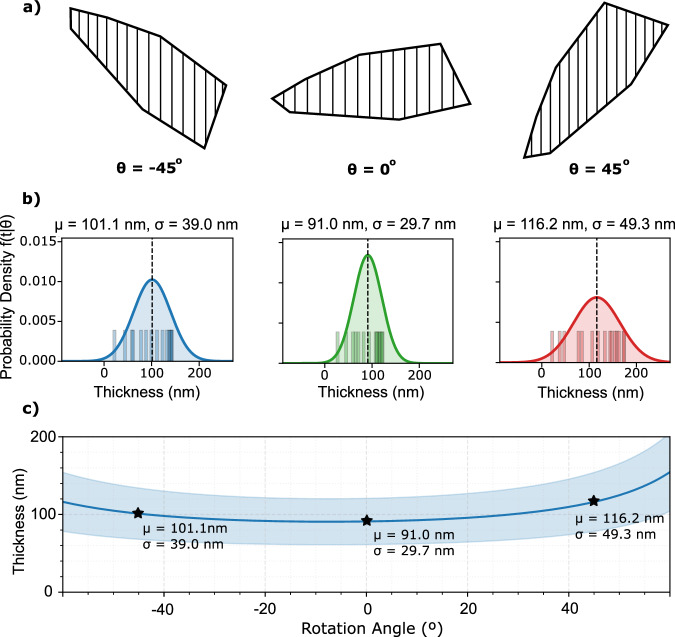
Neural network learning of orientation-dependent crystal thickness distributions. **a** Schematic showing a crystal roughly approximated by a collection of small, parallel slabs with varying thickness at different orientations. **b** Gaussian distributions fit over the distribution of thicknesses for each crystal orientation shown in (**a**). A neural network learns to predict *μ*(*θ*) (the mean) and *σ*(*θ*) (the standard deviation) for each orientation during crystallographic refinement by minimizing *wR*_all_. **c** Plot of representative predicted *μ*(*θ*) and *σ*(*θ*) for a dataset spanning +/- 60^∘^. Stars represent the location of Gaussians plotted in (**b**).

Diffracted intensities are highly sensitive to crystal thickness. Since a typical 3D-ED experiment illuminates a large volume of an irregular crystal simultaneously, the recorded diffraction pattern inevitably integrates signals arising from many variations in crystal thickness across the probed volume. An additional complication is that as the crystal rotates, the projected thicknesses of the sample relative to the diffracted beam change as a function of rotation angle. Previous work has addressed this complexity with a dual pronged approach. First, by invoking the column approximation^36^, the crystal can be simplified into a collection of independent, parallel scattering columns (Fig. 3a). The diffracted intensity can then be treated as an incoherent sum of diffraction patterns emerging from these small columns. To make this calculation tractable, the unknown morphology of the real crystal is approximated with an idealized geometric model such as a wedge, lens, or cylinder from which the required thickness probability density functions can be derived analytically^30^. Secondly, to account for sample rotation, these models are then adjusted by applying empirical corrections that interpolate between two limiting cases of sample geometry: an infinite flat plane, where, for tilt angle *θ*, the apparent thickness varies as $t(\theta )={t}_{0}/\cos \theta$, and a cylindrical or spherical object, where the thickness remains constant, *t*(*θ*) = *t*_0_^28^.

A limitation of this approach is that it relies on rigid geometric priors, which may lack the flexibility to accurately describe the complex, irregular morphologies encountered in real experimental samples. Moreover, current implementations allow the thickness probability distribution to become decoupled from the tilt correction. This can create physical inconsistencies in the refinement if not done with care. For example, using a probability density function derived from a wedge with a tilt correction parameter for a sphere or cylinder is contradictory. In reality, these properties are intrinsically coupled: the underlying 3D shape of the crystal determines both the statistical distribution of thicknesses and how that distribution evolves under rotation. Finally, as these geometric models are rarely optimized jointly with atomic parameters, significant computational effort is often required to identify the correct shape approximation through manual tuning or grid searches outside the main refinement loop.

Here, we instead propose a flexible, data-driven alternative that replaces fixed geometric priors with a lightweight neural network. Rather than assuming a specific crystal shape (e.g., a wedge), our model learns the statistics of the thickness distribution directly from the diffraction data. We assume a Gaussian form for the thickness distribution at each orientation (tilt angle *θ*) as shown in Fig. 3b, whose mean *μ*(*θ*) and standard deviation *σ*(*θ*) are predicted by the neural network. Representative thickness values are then sampled from this distribution and used as input to the dynamical diffraction simulator. The resulting intensities from each different thickness simulation are then averaged to account for the distribution of thicknesses present in the illuminated volume at that orientation. The network operates jointly with the atomic parameter refinement: as the crystallographic refinement progresses, the network weights are updated via backpropagation to minimize the weighted R-factor (*wR*_all_). This allows the model to predict the optimal *μ*(*θ*) and *σ*(*θ*) for every orientation dynamically, adapting readily to complex, irregular morphologies (Fig. 3c). In practice, we find that explicitly sampling from the predicted thickness distribution and averaging the resulting intensities produces refinement *R*-values comparable to those obtained by evaluating the intensity at the mean thickness alone (see Supplementary Note 3 for discussion). We therefore use only the predicted mean thickness in all refinements reported here for computational efficiency. Further implementation details and comparisons with prior approaches are provided in the Methods section.

### Refinement on synthetic data

To evaluate the ability of our refinement workflow to jointly recover both atomic parameters and sample thickness profiles, we first tested our methodology on synthetic 3D-ED datasets. We generated synthetic datasets for three representative structures: paracetamol, CsPbBr_3_, and quartz (see Methods). Each 3D-ED dataset was corrupted with Poisson noise and was assigned a randomly generated thickness profile that varied smoothly as a function of rotation angle *θ*, mimicking realistic variations in apparent crystal thickness during data acquisition.

Initial models for the refinement were created by adding random atomic displacements drawn from a uniform distribution over the range [ − *Δ*, *Δ*] Å, with maximum displacement magnitude of *Δ* = 0.2 Å. Displacements were generated in a symmetry-aware fashion in that atomic positions were perturbed randomly while preserving crystallographic symmetry operations and special Wyckoff positions. Thermal displacement parameters for the starting models were created by scaling the ground truth thermal displacement parameters in proportion to the magnitude of the atomic displacements. Additionally, for CsPbBr_3_, which has ansiotropic thermal displacement parameters, the starting models were initialized with simplified isotropic values to increase the difficulty of the refinement task.

**Fig. 4:**
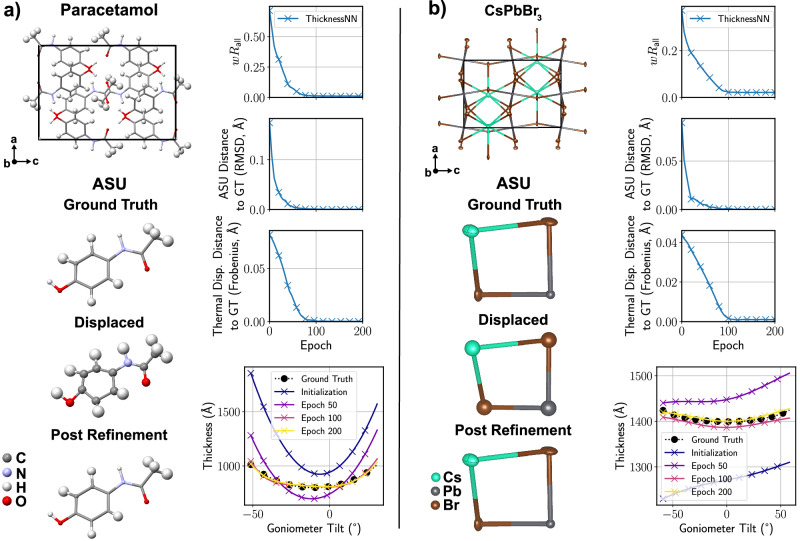
Hybrid Physics-ML model (ThicknessNN) refinements on synthetic data. Refinement results on (**a**) Paracetamol and (**b**) CsPbBr_3_ synthetic 3D-ED datasets. The weighted R-factor (*wR*_all_), the root mean squared distance (RMSD) to ground truth ASU, the Frobenius norm of the difference in thermal displacements, and the thickness distributions are shown as the refinement progresses from initial 0.2 Å randomly displaced starting models. The unit cells, ground truth, starting, and refined models of the asymmetric unit (ASU) are also shown. Thermal displacements are represented by spheres (isotropic) or ellipsoids (anisotropic) centered on atomic sites. The size of the visualized thermal displacements is proportional to their magnitude.

Figure 4 shows representative refinements for CsPbBr_3_ and paracetamol. We observe that *wR*_all_, the root mean squared distance (RMSD) to ground truth atomic positions, and the Frobenius norm (see Methods 4.5) of the difference in thermal displacement parameters all converge effectively to zero within 100 epochs. These results are promising for several reasons. Firstly, a 0.2 Å displacement represents a relatively poor initial model for a crystallographic refinement and we do not employ any stereochemical restraints, yet convergence is still achieved. Secondly, in the cases of CsPbBr_3_, the refinement successfully recovers anisotropic thermal displacements even when initialized with isotropic ones with a large displacement. Thirdly, the ground truth thickness distribution is successfully recovered alongside the atomic parameters and this remarkable performance continues even when the thickness profile has a complex dependence on *θ* (Supplementary Note 1). All of this is achieved using the Adam optimizer^37^ with default settings and without any hyperparameter tuning. Similar results are observed for the quartz datasets (Table 1 and Supplementary Fig. S5), and for models initialised with displacements of 0.1 Å (Supplementary Fig. S4) and 0.3 Å (Supplementary Fig. S6).

**Table 1:** Results comparing refinements with different modeling assumptions (kinematical, constant thickness dynamical, and the ThicknessNN hybrid model) on synthetic 3D-ED datasets

Synthetic Sample	Metric	Kinematical	Dynamical (Single Thickness)	Hybrid Physics-ML (ThicknessNN)
**Quartz**	*wR*_all_	0.480	0.121	0.013
	ASU RMSD (Å)	0.0384	0.00035	0.00067
	Thermal Disp. (Å)	0.0139	0.00045	0.00010
**CsPbBr**_3_	*wR*_all_	0.557	0.126	0.0217
	ASU RMSD (Å)	0.0482	0.0069	0.00040
	Thermal Disp. (Å)	0.0664	0.0145	0.0010
**Paracetamol**	*wR*_all_	0.327	0.0265	0.0112
	ASU RMSD (Å)	0.0163	0.00070	0.00048
	Thermal Disp. (Å)	0.0101	0.00105	0.00025

We report the weighted R-factor (*wR*_all_), the root mean squared distance (RMSD) to ground truth ASU, and the Frobenius norm of the difference in thermal displacements for converged models after refining for 200 epochs from an initial 0.2 Å randomly displaced starting model.

**Fig. 5:**
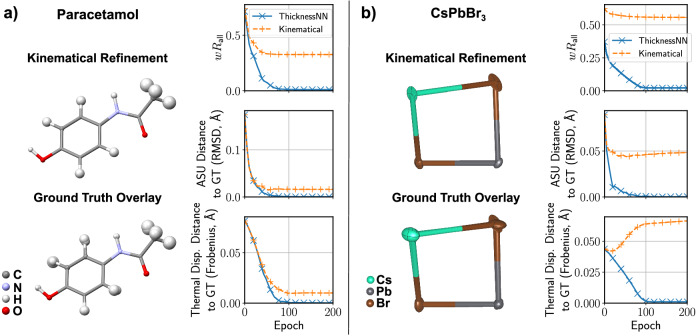
Kinematical refinements on synthetic data. Kinematical refinement results on (**a**) Paracetamol and (**b**) CsPbBr_3_ synthetic 3D-ED datasets. The weighted R-factor (*wR*_all_), the root mean squared distance (RMSD) to ground truth asymmetric unit (ASU), and the Frobenius norm of the difference in thermal displacements are shown alongside the refined ASU and ground truth overlap from initial 0.2 Å randomly displaced starting models. Refinement curves showing the refinement using the proposed hybrid model (ThicknessNN) are also shown for comparison.

To understand the importance of utilising a fully dynamical forward model in the refinement of 3D-ED data, we compare in Fig. 5 our results to those obtained with a kinematical model which we approximate by fixing the sample thickness to 10 Å such that multiple scattering is negligible. As expected, a degradation in refinement quality is observed when using a kinematical model and it is most pronounced when strong dynamical scattering is present in the dataset. For instance, in CsPbBr_3_, where substantial dynamical effects are expected due to the combination of the presence of heavy elements (Cs and Pb) and a large simulated crystal thickness (Fig. 4), the final RMSD in atomic positions and the Frobenius norm (Methods 4.5) of the thermal displacements remain substantially large compared to the dynamical models. In contrast, the performance gap is smaller for paracetamol, which consists of lighter atoms and was simulated with a thinner crystal.

Summary metrics for the converged refinements across all three structures are shown in Table 1. Here we also ablate performance against a dynamical refinement with an imperfect thickness model, where we assume a constant effective thickness for the entire tilt series rather than a detailed thickness profile. The recovered single thickness values along with the ground truth thickness distributions are shown in Supplementary Fig. S3. We find that the single-thickness model also consistently under-performs compared to the hybrid approach. This is especially pronounced in quartz where strong variation with *θ* is present. We observe similar trends for various levels of structural perturbation. Full refinement curves and results across datasets are provided in Supplementary Figs. S4, S5 and S6. We further note runtime comparisons to second order methods in Supplementary Note 2.

### Refinement on experimental data

In the refinement of synthetic datasets, we assume purely elastic scattering with added Poisson noise, and the experimental orientation is considered to be perfectly known. In contrast, real experimental data are affected by additional complexities, including inelastic scattering processes-such as plasmon losses and thermal diffuse scattering^38,39^-as well as detector noise^33^. Accurate frame-by-frame orientation determination is also essential to match simulations to measured intensities^33^. To evaluate the robustness of our proposed hybrid physics-ML refinement approach on real data, we reproduced dynamical refinements for three previously published 3D electron diffraction (3D-ED) datasets: quartz^35^, CsPbBr_3_^28^, and paracetamol^40^. In each case, the original analyses involved data processing using the PETS2 software package^33^, followed by dynamical refinements with DYNGO and JANA^41^.

For our refinements, we used the same PETS2 output files reported in the literature but replaced the DYNGO-JANA pipeline with our own hybrid physics-ML refinement code. To the best of our knowledge, this is the first independent demonstration and benchmarking of an alternative codebase to JANA and DYNGO for dynamical refinement of 3D-ED datasets. Other dynamical refinement codebases^31,42–44^ in the past have focused on the refinement of non-integrated intensities extracted from individual diffraction patterns (often convergent beam electron diffraction; CBED) collected near major crystallographic zone axes, which is a fundamentally different problem to the one presented here. Table 2 summarizes our results. Across all datasets, the number of *N*_obs_ (I  > 3*σ*) and *N*_all_ intensities compares very closely between our refinements and the original studies. The small differences in *N*_obs_ and *N*_all_ between our results and those in the literature likely arise from variations in orientation search protocols and parameterizations. For continuous rotation datasets (quartz and CsPbBr_3_), we perform a three-parameter simplex search involving rotations about the crystal’s *x*, *y*, and *z* axes (see Methods 4.3). In contrast, published studies appear to predominantly use a two-parameter Euler angle approach - *ϕ* (rotation about the laboratory z-axis) and *θ* (tilt about the new x-axis) - for both continuous rotation and precession datasets^34,35^. For precession electron diffraction data (Paracetamol), we follow this convention, where *ϕ* defines the tilt direction and *θ* its amplitude.

**Table 2:** Results of dynamical refinements performed on experimental data

Sample	Metric	DYNGO-JANA	Hybrid Physics-ML
**Quartz**	*R*_obs_	5.7 %^35^	4.2 %
(*Continuous-*	*wR*_all_	6.6 %^35^	4.3 %
*Rotation*)	*N*_obs_, *N*_all_	994, 1710	957, 1734
	${g}_{\max }$ (Å^−1^)	1.6	1.6
**CsPbBr**_3_	*R*_obs_	5.5 %^28^	6.4 %
(*Continuous-*	*wR*_all_	6.8 %^28^	6.7 %
*Rotation*)	*N*_obs_, *N*_all_	2709, 16345	2714, 17095
	${g}_{\max }$ (Å^−1^)	2.0	2.0
**Paracetamol**	*R*_obs_	9.2 %^40^	10.15 %
(*Precession*)	*wR*_all_	10.34 %^40^	8.4 %
	*N*_obs_, *N*_all_	2738^a^, 6669^a^	2697, 6621
	${g}_{\max }$ (Å^−1^)	1.2	1.2

^a^*N*_obs_, *N*_all_ for the paracetamol dataset were not explicitly reported at 1.2 (Å^−1^) resolution. These values were estimated by examining the number of reflections that passed *R*_obs_ and *wR*_all_ filters in the experimental dataset provided in Ref. ^40^.

**Fig. 6:**
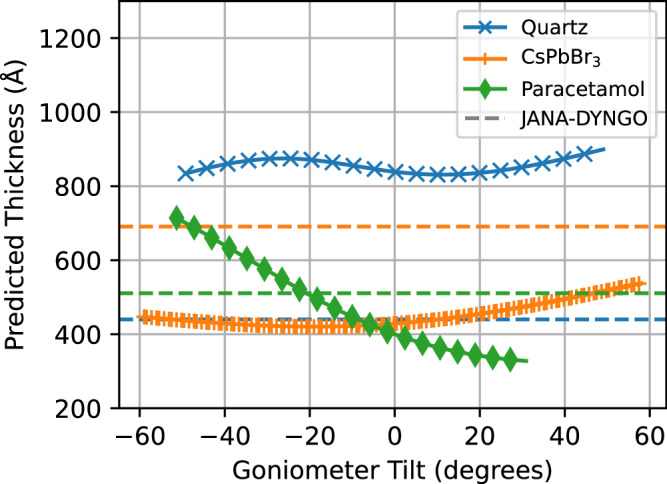
Applying the ThicknessNN to explerimental datasets. Experimental thickness curves recovered from the hybrid physics-ML model and DYNGO-JANA (dashed lines) for Quartz, CsPbBr_3_, and Paracetamol datasets.

For the quartz dataset, the hybrid physics-ML method achieves a lower *R*_obs_ value (4.2%) and *wR*_all_ value (4.3 %) compared to the DYNGO-JANA values of (5.7%) and (6.6%), indicating improved fit to the observed intensities. This is likely due to the thickness modeling employed in the hybrid physics-ML refinement; in Ref. ^35^, from which the quartz dataset is obtained, there is no explicit mention of modeling the thickness of the crystal as a function of *θ* beyond a single refined thickness value of  ~ 44 nm. By comparison, the ThicknessNN utilised in the hybrid physics-ML method recovers a more complex relationship of apparent thickness as a function of *θ* in addition to an overall larger crystal thickness at each orientation (~ 85 nm average). The discrepancy between these thickness values is difficult to explain. Notably though, if we turn off the ThicknessNN and use a single thickness value in our simulator, we observe a minimum in *wR*_all_ at 87.2 nm (Supplementary Fig. S8) which compares very closely with the mean thickness recovered by the ThicknessNN. Additionally, interrogating images of the crystal from which the 3D-ED dataset is recorded – though a qualitative analysis at best – suggests that the true mean crystal thickness is likely closer to 85 nm than 44 nm (See Supplementary Fig. S9 and Supplementary Note 4). The recovered thickness distribution (Fig. 6) is plausible given the complex geometry of the quartz crystal and possible specimen movement during data acquisition (See Supplementary Video 1 and Supplementary Note 5). However, we note that it is difficult to derive accurate 3D specimen geometries from experimental electron microscopy images without a full tomographic analysis.

For CsPbBr_3_, the recovered *R*_obs_ and *wR*_all_ values are comparable between our hybrid Physics-ML model (6.4% *R*_obs_, 6.7% *wR*_all_) and those reported in the original study (5.5% *R*_obs_, 6.8% *wR*_all_)^28^. These similar residuals are expected given that both approaches incorporate an explicit thickness model. However, the underlying recovered thickness distributions differ. The original study reports a constant thickness of 69.1 nm across the dataset and noted that introducing a tilt correction parameter-intended to vary thickness as a function of *θ* - did not improve the residuals. This suggests a sample geometry approximating a uniform cylindrical rod, such that *t*(*θ*) = *t*_0_. In contrast, our model recovers a thickness distribution with a mean of 44.0 nm and a mild dependence on *θ* (see Fig. 6). An advantage of our approach is that we do not require an explicit tilt correction parameter, which in the original study may have necessitated multiple refinement cycles to identify an optimal form. In this case, both models yield similar residuals because the thickness distribution is relatively simple. However, in more complex scenarios-such as the quartz dataset discussed earlier-a manually defined tilt correction function would likely fail to capture the true variation, highlighting the flexibility and generality of our approach.

For the paracetamol dataset, our *R*_obs_ is higher than reported (10.15% vs 9.2%), but our *wR*_all_ is lower (8.4% vs 10.34%), which is atypical, but has been observed in other dynamical refinements that utilise the same weighting in the calculation of *wR*_all_ (see Methods)^34^. The recovered thickness distribution with the hybrid physics-ML model is asymmetric, which is roughly consistent with the rotation range covered by the goniometer during the experiments (-52^∘^ to +30^∘^), or alternatively, could be explained by the electron beam intentionally being moved across the sample during acquisition to mitigate beam damage as previously reported^21^. Interestingly, the mean value of thickness distribution recovered from the hybrid physics-ML model (47.5 nm) compares closely to the single thickness value refined with the DYNGO-JANA model (51.1 nm^40^, Fig. 6).

One of the key advantages of electron diffraction is its high sensitivity to the chirality (handedness) of non-centrosymmetric crystal structures. In such systems, chirality is encoded in the violation of Friedel’s Law, which results in intensity differences between symmetry-related reflections **g** and  − **g**. This asymmetry arises from multiple scattering effects and cannot be interpreted using the kinematical approximation alone. Instead, full dynamical simulations are required, involving refinements of both enantiomorphic models to determine the correct absolute structure^26,35^. To verify that our hybrid physics-ML model can recover these differences we perform independent refinements of both enantiomers (*P*3_2_21 and *P*3_1_21) of quartz to replicate the results presented in Ref. ^35^. We obtain substantially lower residual factors with *P*3_2_21 enantiomer (Table 3), which is consistent with previous results^35^ and demonstrates that the hybrid physics-ML model is capable of accurately determining the absolute structure of chiral systems.

**Table 3:** Absolute structure determination from the 3D-ED quartz dataset

Sample	Enantiomorph 1	Enantiomorph 2
**Quartz**		
Spacegroup	*P*3_2_21	*P*3_1_21
${g}_{\max }$ (Å^−1^)	1.6	1.6
*N*_obs_, *N*_all_	957, 1734	957, 1734
*wR*_all_	4.3 %	7.1 %
*R*_obs_	4.2 %	6.2 %

## Discussion

Our results demonstrate that hybrid physics-machine learning models, built on a foundation of differentiable physics, can offer a powerful framework for the dynamical refinement of 3D electron diffraction data. As a proof of principle, we have focused here on the application of this framework to modeling crystal thickness variations. Our results on both synthetic and experimental data are encouraging. In the case of synthetic data, recovery of ground truth thickness is almost flawless suggesting that it could someday be possible to eliminate thickness variations as an uncertainty in experimental refinements. While a complete evaluation of this hypothesis was not possible with the experimental data utilised in this study, future work that is performed on data extracted from experimental specimens of known geometry, such as those obtained from focused ion beam milling, will enable a rigorous assessment of how closely our framework can recover ground-truth thickness distributions.

Future work can also explore the applicability of this framework to other sources of error within existing refinement protocols. For example, models – neural network based or otherwise – of beam damage, inelastic scattering or detector noise that are physically constrained to avoid overfitting could be developed. Incorporating such physical phenomena into the refinement process would further lower residual *R*-factors while simultaneously yielding dataset-specific estimates of, for example, beam damage and inelastic scattering.

An even more compelling prospect is the potential for this framework to serve as a development base for robust quantum electron crystallography. In our Bloch wave forward model, as supposedly in greater than 99% of crystallographic refinements^45^, we implicitly assume that each atom is described by a neutral, spherically symmetric, charge density centered on the atomic nucleus. This approximation is known as the independent atom model. In real materials and molecules, chemical bonding between atoms leads to deformations of charge density — and thus electrostatic potential – that are directly measurable with electron scattering^28,43,44^. However, quantifying these features remains non-trivial. Approaches such as multipole modeling and Hirshfeld atom refinement, originally developed for X-ray crystallography, have shown promising potential for 3D-ED refinements^28,46,47^ but further development of these frameworks, and others, will be required to determine which physical descriptions are most appropriate for dynamical electron scattering.

Regardless of the specific physical model chosen, the differentiable nature of the framework presented here significantly lowers the barrier to entry for future developments, as it eliminates the need to analytically derive gradients for complex descriptions of the scattering potential, and naturally accommodates future adoption of hybrid physics-machine learned representations of the electrostatic potential.

Finally, while this work focused on 3D-ED using the Bloch wave formalism, the paradigm of differentiable hybrid modeling is broadly applicable. Similar hybrid physics-ML frameworks could readily be adapted to other simulation methods, such as the multislice algorithm, and extended to diverse modalities like 4D scanning transmission electron microscopy (4D-STEM), ultimately providing an updated standard for quantitative analysis across the electron microscopy community.

## Methods

### Hybrid physics-ML model

Our forward diffraction model, shown in Fig. 2, consists of a hybrid neural-network based thickness model and a differentiable physics simulator, combining the physical accuracy of the Bloch wave formalism and the flexibility of neural network function approximation.

#### Differentiable bloch wave propagation

The Bloch wave method, developed by Bethe^29^ and later expanded by others^19,25,36^, models elastic electron scattering in perfect crystals across arbitrary orientations and thicknesses, yielding diffracted intensities that can be directly compared to 3D-ED data. Its matrix formulation is naturally differentiable, making it well adapted to gradient-based optimization in dynamical refinement^34^. The Bloch wave formalism expands the wavefunction of the incident electron *ψ*(**r**) into a finite set of plane-wave states permitted by the periodic crystal potential *V*(**r**). For a complete theoretical treatment, see Spence and Zuo^48^. The periodic crystal potential is expressed as *V*(**r**) = ∑_**g**_*V*_**g**_e^2*πi***g**⋅**r**^, where each reciprocal lattice vector **g** corresponds to a Fourier component *V*_**g**_, determined by the underlying atomic model.

To compute *V*_**g**_, the ASU is expanded to the full unit cell using the crystal’s space group operations. Each atom *i* contributes an electron scattering factor ${f}_{i}^{e}$, evaluated at scattering vector $s=| \frac{g}{2}|$, modulated by the Debye-Waller factor *B*_*i*_ that accounts for atomic thermal motion. *V*_**g**_ then takes the form:

1 $${V}_{{{{\bf{g}}}}}=\frac{1}{\Omega }{\sum }_{i}{f}_{i}^{e}({{{\bf{s}}}})\,\exp \left(-{B}_{i}{s}^{2}\right)\,\exp (-2\pi i{{{\bf{g}}}}\cdot {{{{\bf{r}}}}}_{i}),$$

where Ω is the unit cell volume and **r**_*i*_ is the atomic position. Thermal vibrations reduce the sharpness of the periodic potential, leading to overall damping of diffracted intensities, with stronger attenuation at higher scattering angles. For isotropic displacements, the Debye-Waller factor is given by ${B}_{i}=8{\pi }^{2}\langle {u}_{i}^{2}\rangle$, where $\langle {u}_{i}^{2}\rangle$ is the Cartesian atom-specific mean-square displacement. In the anisotropic case, thermal motion is described by a symmetric second-rank tensor *U*_*ij*_, defined as the covariance *U*_*ij*_ = 〈*u*_*i*_*u*_*j*_〉 of atomic displacements along Cartesian directions *i* and *j*^49^. To express *U*_*ij*_ in crystallographic fractional coordinates, it is transformed using the orthogonalization matrix *A*:

2 $${U}_{ij}^{\star }={A}^{-1}{U}_{ij}^{{{{\rm{cart}}}}}{A}^{-{\mathsf{T}}}$$

This transformation ensures compatibility with space group symmetry operations. To maintain physical validity and enable stable refinement, the tensor is parameterized via its Cholesky decomposition *U* = *LL*^⊤^, where *L* is a lower triangular matrix. This guarantees positive-definiteness and enables smooth optimization. During asymmetric unit expansion, each ${U}_{ij}^{\star }$ is rotated under the space group operations and then converted back to Cartesian form for use in structure factor calculations.

#### Apparent thickness neural network

To model orientation-dependent variations in apparent crystal thickness, we implemented a lightweight neural network module (ThicknessNN) in PyTorch^17^ that maps a rotation angle *θ* to a predicted mean thickness *μ*(*θ*) and associated standard deviation *σ*(*θ*). The model consists of a feedforward architecture with two hidden layers of 64 units each and Tanh activation functions (to promote smoothness in *θ*), followed by a two-node output layer that returns *μ* and $\log {\sigma }^{2}$. The predicted standard deviation is scaled to lie within a user-defined range to ensure numerical stability.

Users may often have prior knowledge of both the approximate thickness range (e.g. 10-200 nm) and the qualitative shape of its variation across orientations. Such priors can be incorporated to warm-start the optimization. For example, we incorporate an optional quadratic prior by expressing *μ*(*θ*) as a weighted combination of a learnable quadratic function *aθ*^2^ + *bθ* + *c* (with *a* > 0 enforced via exponential parameterization) and a residual term learned from the network. This hybrid formulation allows the model to learn deviations from simple geometric thickness profiles while retaining regularizing inductive structure. In synthetic data experiments, we utilized this prior for quartz and paracetamol, however we soon realised that it was not required for reliable thickness distribution recovery. For subsequent experiments including synthetic CsPbBr_3_ and experimental data, we did not add this prior, though we note in particular high-noise or data scarce environments (such as refining on partial datasets), these priors may prove useful.

##### General Framework

For a reflection *h*, the diffracted intensity at a given crystal thickness *t* is denoted *I*_*h*_(*t*). If the illuminated region contains a distribution of thicknesses, the observed intensity corresponds to the expectation of *I*_*h*_(*t*) over the thickness probability distribution function (PDF) *f*(*t*):

3 $${I}_{h}^{\,{{{\rm{obs}}}}}=\int \,{I}_{h}(t)\,f(t)\,dt,\,\int \,f(t)\,dt=1.$$

Both the present method and earlier approaches^30^ are based on this same principle, differing primarily in the parameterisation of *f*(*t*). Previous approaches typically derived *f*(*t*) from assumed *geometric priors* (e.g. wedge, cylindrical, ribbon, or convex-lens morphologies), from which closed-form cumulative thickness distributions could be obtained. Tilt dependence was often introduced through empirical corrections that adjusted the mean thickness as a function of orientation.

##### Neural Net Parameterisation

In contrast, our approach replaces the fixed geometric parameterisation with a data-driven parameterisation of the thickness probability density function. At each orientation (tilt angle *θ*), we assume a Gaussian form for the thickness distribution *f*(*t*∣*θ*), whose mean *μ*(*θ*) and standard deviation *σ*(*θ*) are predicted by a neural network:

4 $$f(t| \theta )=\frac{1}{\sigma (\theta )\sqrt{2\pi }}\exp \left[-\frac{{(t-\mu (\theta ))}^{2}}{2\sigma {(\theta )}^{2}}\right].$$

This formulation enables the thickness distribution to adapt smoothly to complex, non-geometric variations in the specimen while remaining differentiable with respect to the distribution parameters *μ*(*θ*) and *σ*(*θ*). No manual tuning of empirical parameters is required, as the network learns the distribution directly from the experimental data.

##### Computation

To compute the expected intensity, we use the reparameterization trick to generate differentiable samples analogously to variational autoencoders^50^:

5 $$t=\mu (\theta )+\sigma (\theta )\,\epsilon,\,\epsilon \sim {{{\mathcal{N}}}}(0,1),$$

and approximate the expectation as

6 $${I}_{h}^{\,{{{\rm{obs}}}}}(\theta )\approx \frac{1}{N}{\sum }_{j=1}^{N}{I}_{h}\,\left(\mu (\theta )+\sigma (\theta )\,{\epsilon }_{j}\right).$$

This enables gradients to propagate through both *μ*(*θ*) and *σ*(*θ*) during refinement, allowing the model to learn orientation-dependent thickness statistics directly from data.

##### Simplified Evaluation

In practice, we found that sampling from the predicted Gaussian and averaging the resulting intensities yielded similar refinement *R*-values to simply evaluating the intensity at the mean thickness *μ*(*θ*) (Supplementary Note 3). For computational efficiency, all refinements reported in this work therefore used

7 $${I}_{h}^{\,{{{\rm{obs}}}}}(\theta )\approx {I}_{h}\,\left(\mu (\theta )\right).$$

Nevertheless, the framework remains fully compatible with explicit distributional averaging and could be extended to more flexible, non-Gaussian formulations if required in future applications.

### Refinements, loss functions and reflection filtering

For calculation of residuals *wR*_all_ and *R*_obs_ we followed the conventions and definitions outlined in Klar et al.^35^.

8 $$R=\frac{\sum \left|\sqrt{{I}_{{{{\rm{obs}}}}}}-\sqrt{{I}_{{{{\rm{calc}}}}}}\right|}{\sum \sqrt{{I}_{{{{\rm{obs}}}}}}}$$

9 $$wR=\sqrt{\frac{\sum {\left(w\left|{I}_{{{{\rm{obs}}}}}-{I}_{{{{\rm{calc}}}}}\right|\right)}^{2}}{\sum {\left(w{I}_{{{{\rm{obs}}}}}\right)}^{2}}}$$

10 $$w={\left(\sigma {\left(\sqrt{{I}_{{{{\rm{obs}}}}}}\right)}^{2}+{\left(u\sqrt{{I}_{{{{\rm{obs}}}}}}\right)}^{2}\right)}^{-1/2}$$

Where the sums run over all reflections for the calculation of *wR*_all_, and only over observed reflections with *I*_obs_ > 3*σ*(*I*_obs_) for *R*_obs_. The instability factor *u* was set to 0.01. Reflections with either weak or negative intensities, with *I*_obs_ < 0.01 *σ*(*I*_obs_) were set to 0, and their uncertainties were adjusted such that

11 $$\sigma \left(\sqrt{{I}_{{{{\rm{obs}}}}}}\right)=5\sqrt{\sigma ({I}_{{{{\rm{obs}}}}})}.$$

All refinements of atomic positions, anisotropic thermal displacement, ThicknessNN weights and biases and orientations proceeded against *wR*_all_ and did not utilise any restraints or constraints beyond those imposed by crystal symmetry for a particular spacegroup. For all experiments, we refined parameters using the Adam optimizer^37^ over 200 epochs (passes over the full dataset of rotations) using a full batch gradient descent (we average the loss over all rotations for each gradient step), and a learning rate of 0.001 for all parameters.

For selection of integrated intensities, the methodologies to calculate *S*_*g*_, ${S}_{g}^{max}$, *D*_*sg*_ and *R*_*sg*_ described in Ref. ^35^ were again followed. Values for *S*_*g*_, ${S}_{g}^{max}$, *D*_*sg*_ and *R*_*sg*_ utilized in the refinements of experimental data were as previously reported^28,35,40^.

### Orientation refinement

Orientation refinement for each rotation was performed using either a three-parameter Nelder-Mead simplex search or a modified simplex approach based on the methodology described by Palatinus et al.^34^. In the Nelder-Mead approach, the crystal orientation was parameterized as a product of three small-angle rotations about the crystal’s *x*, *y*, and *z* axes, with the optimization objective being the minimization of the residual *wR*_all_. The three rotation angles *α*, *β*, and *ω* were optimized using the scipy.optimize.minimize function with method='Nelder-Mead', initialized from a regular simplex^51^. At each optimization step, an updated orientation matrix was constructed and used in the forward simulation of dynamical diffraction intensities. The updated orientation matrix had the form

12 $$R={R}_{z}(\omega ){R}_{y}(\beta ){R}_{x}(\alpha )$$

The alternative two parameter orientation search followed a method whereby a discrete hexagonal scan over orientations defined by two angular parameters: (*ϕ*), a rotation about the laboratory *z*-axis, and *θ*, a tilt about the rotated *x*-axis was performed. Trial orientations were generated by rotating the current orientation matrix as

13 $${R}_{{{{\rm{new}}}}}={R}_{z}(\phi ){R}_{x}(\theta ){R}_{z}(-\phi ){R}_{{{{\rm{current}}}}},$$

with (*ϕ*) varied in 60^∘^ increments and *θ* halved iteratively if no improvement in *wR*_all_ was found. This approach effectively performs a simplex-like local search over a cone of directions around the current orientation.

Both search protocols used the full experimental-simulated diffraction matching pipeline described above, including filtering of reflections and scaling factor optimization, to evaluate the objective function at each orientation step.

### Synthetic data generation and noise addition

The synthetic data utilised in this study was generated from atomic co-ordinates obtained from successful experimental dynamical refinements of quartz^21,28^.

These three structures were chosen as they span many of the current application areas of dynamical refinements of 3D-ED data (inorganic materials science and small molecule crystallography) and will exhibit varying degrees of dynamical effects. For example. we expect CsPbBr_3_ to have more dynamical scattering than paracetamol for similar thicknesses.

To model experimental noise in simulated diffraction data, we applied Poisson-distributed noise across the full dataset. All simulated intensities were first linearly scaled such that the brightest reflection across the dataset matched a predefined dynamic range maximum (e.g. ${I}_{\max }$ = 10^6^ or ${I}_{\max }$ = 10^2^ etc). This ensures control over the signal-to-noise ratio (SNR), with lower dynamic range values producing proportionally higher noise relative to signal, thereby emulating low-dose data acquisition conditions. After scaling, reflections were removed from each individual pattern if their intensities were more than a factor of the dynamic range below the global maximum, i.e., if their scaled intensity fell below one count. This step mimics a practical detector noise floor and enforces the selected dynamic range as a hard cutoff on observable intensities. Poisson noise was then applied to the remaining scaled intensities using the torch.poisson function from the PyTorch library, which samples from a Poisson distribution with mean equal to the scaled intensity. This implementation captures counting statistics typical of electron diffraction experiments while preserving the statistical structure of the dataset. The synthetic data presented in the main text was generated using a dynamic range of 10^6^, consistent with the capabilities of state-of-the-art photon-counting detectors. The methodology was also evaluated under high-noise conditions using a reduced dynamic range of 10^2^ to assess robustness in low-SNR regimes (Supplementary Fig. S2). Uncertainties for each reflection were estimated as the square root of the noisy intensities, i.e., ${\sigma }_{i}=\sqrt{{I}_{i}}$, consistent with Poisson counting statistics.

### Frobenius norm

For a matrix ${{{\bf{A}}}}\in {{\mathbb{R}}}^{m\times n}$, the Frobenius norm is defined as

14 $$\parallel {{{\bf{A}}}}{\parallel }_{F}={\left({\sum }_{i=1}^{m}{\sum }_{j=1}^{n}| {a}_{ij}{| }^{2}\right)}^{1/2}.$$

In this work, the Frobenius norm is used to quantify the distance of the refined thermal displacement parameter matrices (${{{{\bf{U}}}}}_{{{{\rm{refined}}}}}\in {{\mathbb{R}}}^{3\times 3}$) from their ground-truth values (${{{{\bf{U}}}}}_{{{{\rm{groundtruth}}}}}\in {{\mathbb{R}}}^{3\times 3}$) during crystallographic refinements. We compute and report the average ∥**U**_deviation_∥_*F*_ = ∥**U**_ground truth_ − **U**_refined_∥_*F*_ across atoms in all tables and plots.

### Code development

The code is developed in python and is built on Bloch Wave code originally developed by the creators of abTEM^52^.

## Supplementary information

Supplementary Information

Description of Additional Supplementary Files

Supplementary Video 1

Transparent Peer Review file

## Source data

Source Data

## Data Availability

Relevant data generated during and supporting the findings of this study are available at the following Zenodo link: 10.5281/zenodo.18281349. Source data are provided with this paper.

## Code availability

A version of the codebase suitable for reproducing the results in this paper has been deposited at the following Zenodo link: 10.5281/zenodo.18281349. The authors are collaborating with software developers to make a more stable and mature version of the codebase available for general release on github.
