# Supplementary material for: Hybrid physics-machine learning models for quantitative electron diffraction refinements
Source: Nat Commun. 2026 Apr 11;17:5056. doi: 10.1038/s41467-026-71673-9 (PMC13243467; doi:10.1038/s41467-026-71673-9)
Supplement: Supplementary file 1 — Supplementary Information [file 41467_2026_71673_MOESM1_ESM.pdf]

Supplementary Information for: Hybrid Physics-Machine Learning Models for Quantitative Electron Diffraction Refinements

Shreshth A. Malik[1†], Tiarnan A.S. Doherty[1,2*†], Benjamin Colmey[2], Stephen J. Roberts[3], Yarin Gal[1*], Paul A. Midgley[2*]

[1]OATML, Department of Computer Science, University of Oxford, Wolfson Building, Parks Rd, Oxford, OX1 3QG, United Kingdom.
[2]Department of Materials Science and Metallurgy, University of Cambridge, 27 Charles Babbage Rd, Cambridge, CB3 0FS, United Kingdom.
[3]MLRG, Department of Engineering Science, University of Oxford, Eagle House, Walton Well Road, Oxford, OX2 6ED, United Kingdom.

*Corresponding author(s). E-mail(s): td404@cam.ac.uk; yarin.gal@cs.ox.ac.uk; pam33@cam.ac.uk;
†These authors contributed equally to this work.

Supplementary Note 1: Recovery of Arbitrary Thickness Profiles

To demonstrate the recovery of arbitrary thickness distributions, we test our method on a synthetic 3D-ED dataset of a quartz crystal corrupted with Poisson noise (see Methods). The ground truth thickness distribution varies non-linearly with θ in a sine-like fashion. Remarkably, over 200 epochs of training, the ThicknessNN recovers the full underlying distribution directly from the data, without relying on any prior assumptions about the sample geometry (Figure S1). The only inductive bias imposed, in this case, is a smoothness constraint through the use of Tanh activations, allowing the model to flexibly learn arbitrary variations as a function of θ (see Methods). This highlights the model's capacity to fit complex, non-convex thickness profiles purely through gradient-based refinement. By contrast, existing parametric models, which

seem restricted to recover convex functions (e.g., cosine-like) that are symmetric about $\theta = 0$ or assume constant thickness [1], would not be able to recover this thickness distribution.

We find that our method also accurately recovers thickness distributions in high noise limits (Figure S2).

Supplementary Note 2: Comparing First and Second Order Optimization

To assess the efficiency of the first-order optimization approach utilizing the Adam optimizer [2], which is presented in the main text, we compared runtimes against second-order optimizers such as L-BFGS [3]. The results are summarized in Figure S7. For example, on the $CsPbBr_3$ structure with a 0.1 Å displacement, the refinement using Adam achieves comparable or better accuracy with $\sim 5\times$ speed-up to reach a wR_{all} of ~ 0.03. Adam compares similarly or more efficient than L-BFGS while retaining performance across all synthetic experiments performed. We note however that our implementation has not been heavily optimized, and further quantitative benchmarks and comparisons could be explored in future work.

Supplementary Note 3: Thickness Representation Ablation on Experimental Data

In Section 2.2 and 4.1.2, we highlight that although the proposed ThicknessNN predicts a Gaussian predictive distribution over thickness for a given tilt, in practice we find that using only the predicted mean thickness yields comparable refinement quality with improved computational efficiency. To support this observation, we compared mean-only predictions against sampling from the learned Gaussian distribution on the quartz experimental dataset, using $K = 50$ thickness samples while keeping all other hyperparameters fixed. Sampling from the full predictive distribution resulted in a wR_{all} of 4.64% and an R_{obs} of 4.44%, which is slightly worse than the corresponding results obtained using only the mean prediction (Table 2). Given that training and inference with full distributional sampling increases the computational cost of refinement, we decided to adopt mean-only predictions for all experimental results presented in this work, which is a proof of concept. We emphasize, however, that this finding may reflect data quality in the experimental data, where the additional uncertainty captured by the predictive distribution does not translate into improved refinement performance. In other scenarios explicitly sampling from the predictive distribution may prove beneficial or even necessary. Furthermore, while Gaussian distributions provide a reasonable and convenient approximation for the thickness uncertainty in our current setting, they may be insufficient in cases where the underlying thickness distribution is multimodal or exhibits significant non-Gaussian structure. Importantly, alternative distributional parameterizations could be readily incorporated into our framework in future work without changing the overall refinement pipeline.

Supplementary Note 4: Quartz Experimental Data Thickness

In Section 2.4, Figure 6 we compare thickness profiles obtained from DYNGO-JANA and ThicknessNN. For the quartz dataset we find significant discrepancy between the two methods. One possible explanation for the discrepancy could be that the quartz dataset exhibits relatively weak dynamical effects, such that the diffracted intensities depend only weakly on thickness over a broad range. To investigate this, we conducted a grid search over single thickness values in the range 5 to 2000 Å in 2 Å increments using our Bloch wave simulator. We plot the diffraction intensity loss as a function of thickness in Figure S8. We find a clear single minima at around 870 Å, demonstrating 1) close consistency with the values recovered from our own ThicknessNN 2) clearly observable dynamical effects meaning that the discrepancy cannot be described due to 'kinematical like' dataset alone.

Supplementary Note 5: Quartz thickness profile

The supplementary video associated with the quartz dataset is reconstructed from TEM images [4] acquired during a crystal tracking experiment reported by [5]. In this experiment, a limited number of TEM images were recorded across the experimental tilt range and used to interpolate the expected motion of the crystal during diffraction data acquisition.

Diffraction data were likely collected using a selected area aperture, which will remove scattering from adjacent crystals, and the beam position was adapted to follow the expected crystal position during tilting, as described in the original publication. An image of the illuminated probe after aperture insertion is shown in Supplementary Figure S10.

Supplementary Figures

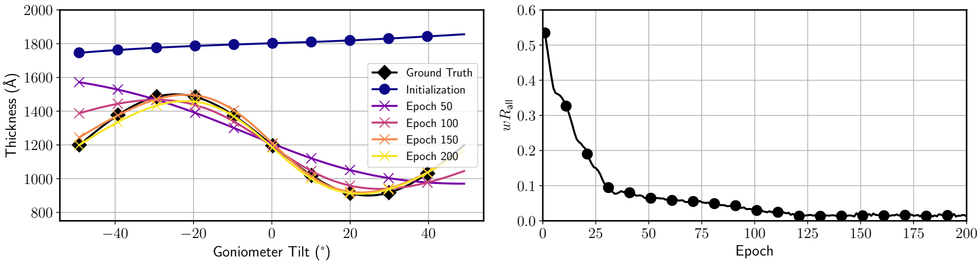

Fig. S1 Refinement of a sinusoidal synthetic thickness distribution using the thickness neural network. The network recovers the true underlying thickness distribution (left) via minimizing the diffraction loss (right).

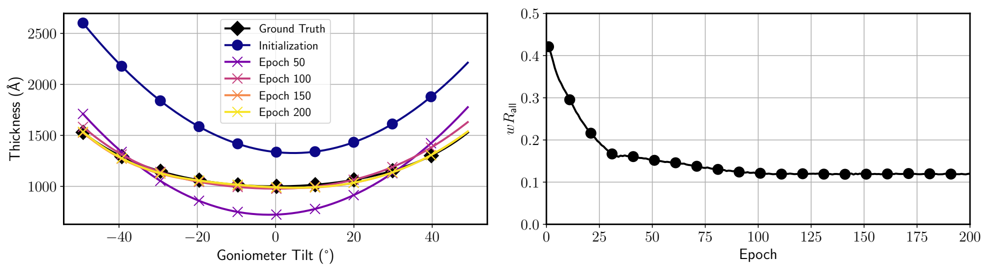

Fig. S2 Successful thickness recovery in a quartz dataset simulated with high noise. The total dynamic range of the diffraction patterns was set to 10^2.

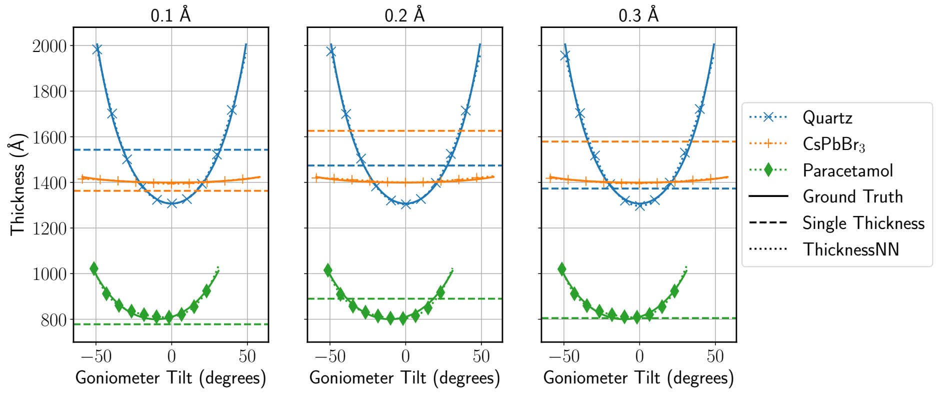

Fig. S3 Ground truth synthetic thicknesses and optimized thicknesses for each displacement, comparing ThicknessNN and a single dynamical thickness. The ground truth and ThicknessNN recovered distributions are overlapping. The single dynamical thickness was found by taking the mean over the optimal thicknesses for each rotation. The optimal thickness for each rotation was found via conducting a grid search from 5 Å to 2000 Å in 2 Å increments to minimize wR_{all}.

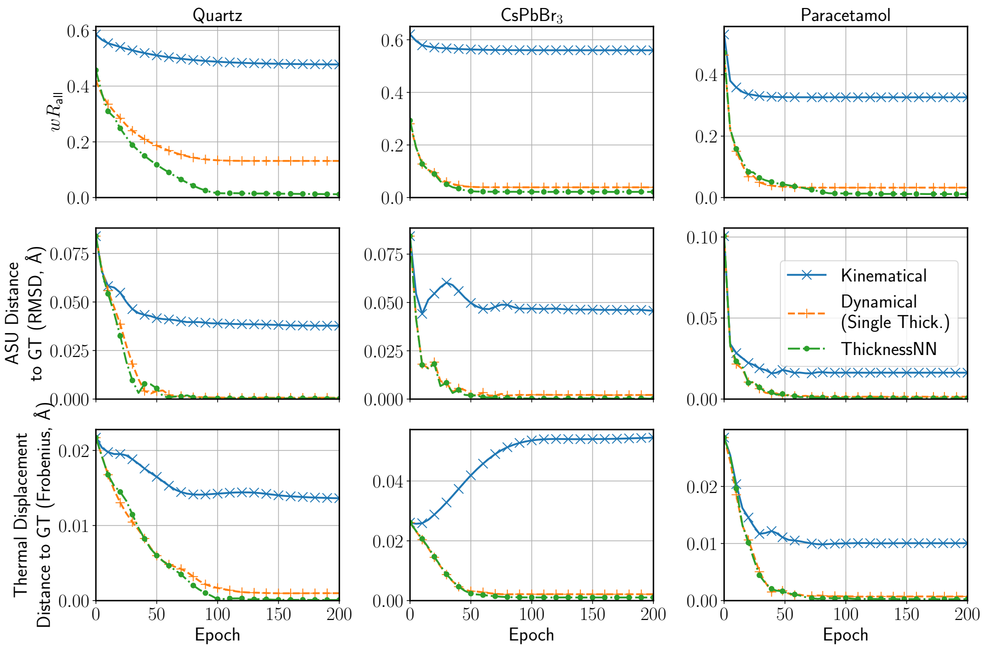

Fig. S4 Refinement of synthetic crystal structures starting from 0.1 Å random maximum displacement from the ground truth. The mean diffraction intensity loss across all rotations in the dataset wR_{all}, the root mean squared displacement (RMSD) to the unperturbed asymmetric unit, and the Frobenium norm of difference in thermal displacement parameters are shown for each material as a function of the refinement time in epochs.

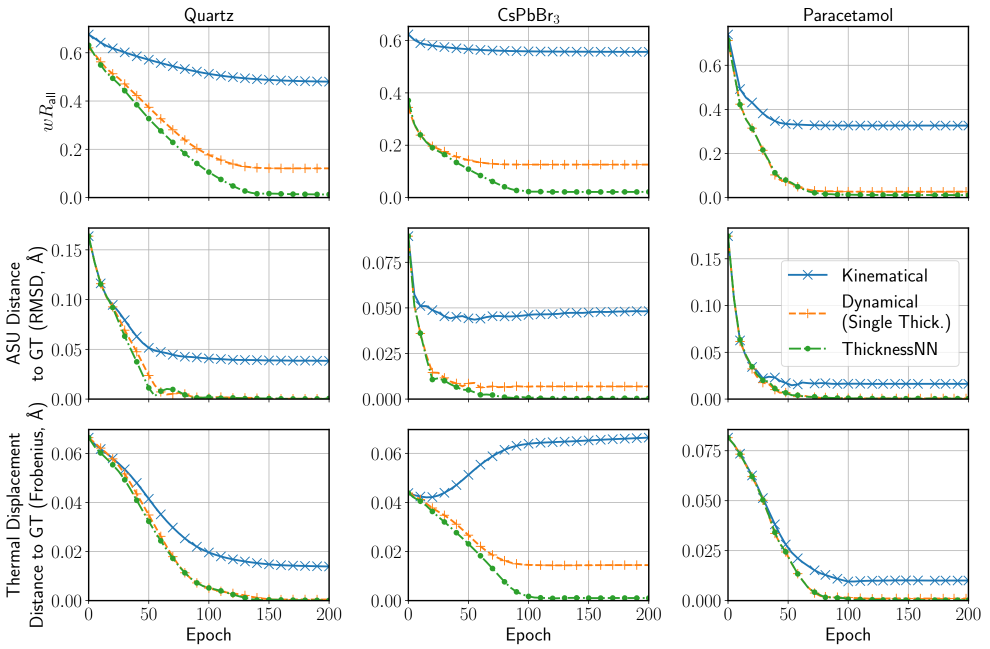

Fig. S5 Refinement of synthetic crystal structures starting from 0.2 Å random maximum displacement from the ground truth. The mean diffraction intensity loss across all rotations in the dataset wR_{all}, the root mean squared displacement (RMSD) to the unperturbed asymmetric unit, and the Frobenium norm of difference in thermal displacement parameters are shown for each material as a function of the refinement time in epochs.

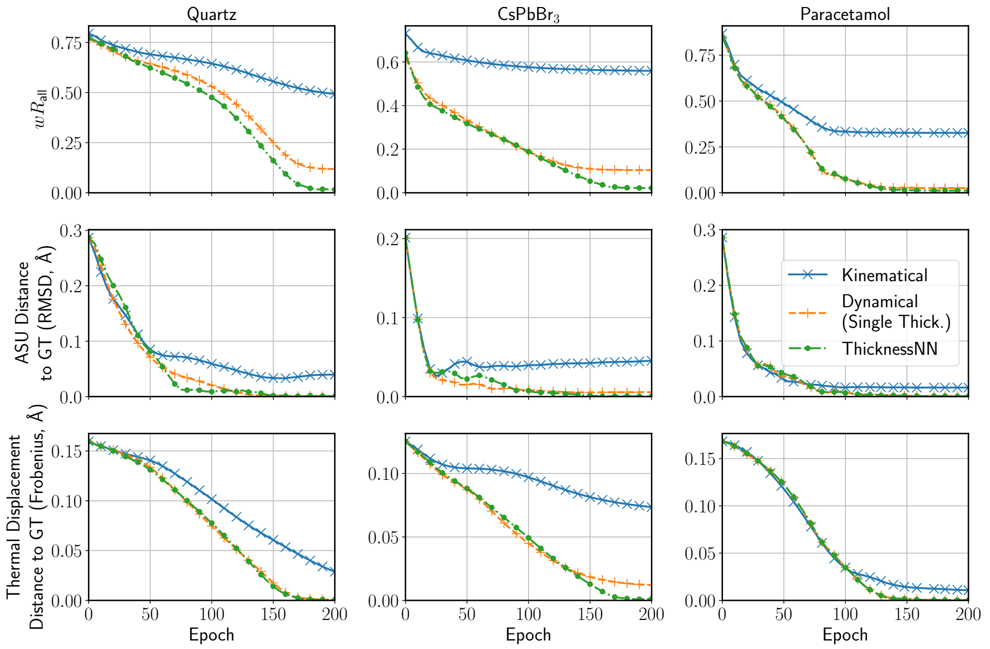

Fig. S6 Refinement of synthetic crystal structures starting from 0.3 Å random maximum displacement from the ground truth. The mean diffraction intensity loss across all rotations in the dataset wR_{all}, the root mean squared displacement (RMSD) to the unperturbed asymmetric unit, and the Frobenium norm of difference in thermal displacement parameters are shown for each material as a function of the refinement time in epochs.

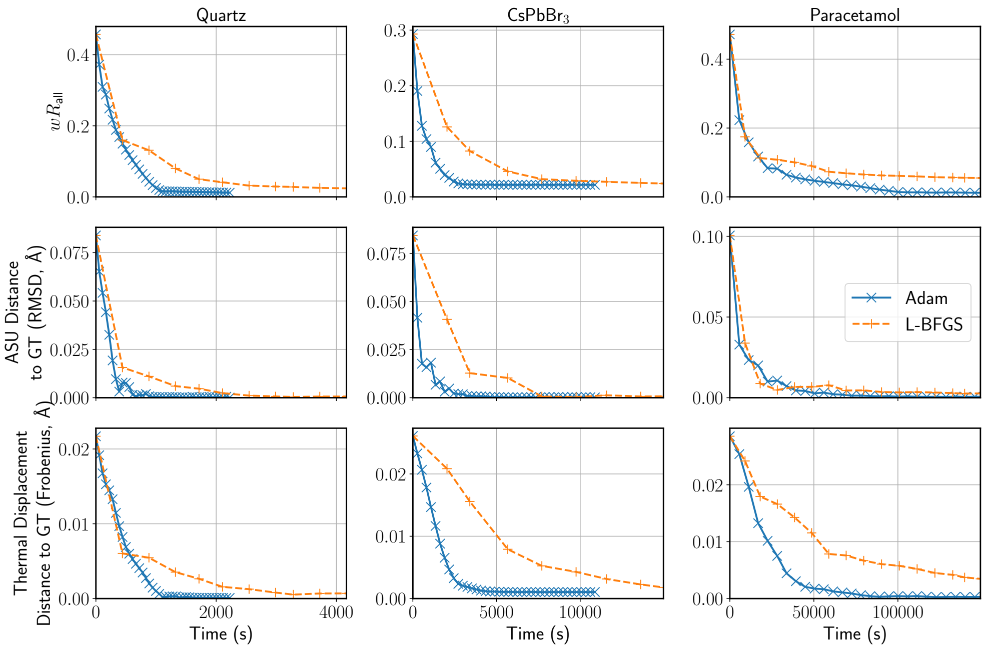

Fig. S7 Refinement of synthetic crystal structures starting from 0.1 Å random maximum displacement from the ground truth, comparing Adam and L-BFGS optimization. The mean diffraction intensity loss across all rotations in the dataset wR_{all}, the root mean squared displacement (RMSD) to the unperturbed asymmetric unit, and the Frobenium norm of difference in thermal displacement parameters are shown for each material as a function of the refinement runtime.

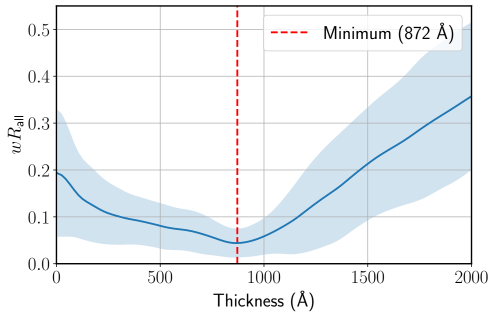

Fig. S8 Diffraction intensity loss as a function of thickness for the quartz experimental dataset. The shaded area indicates the standard deviation of the loss over all the rotations in the dataset. We find a single minima at around 870 Å.

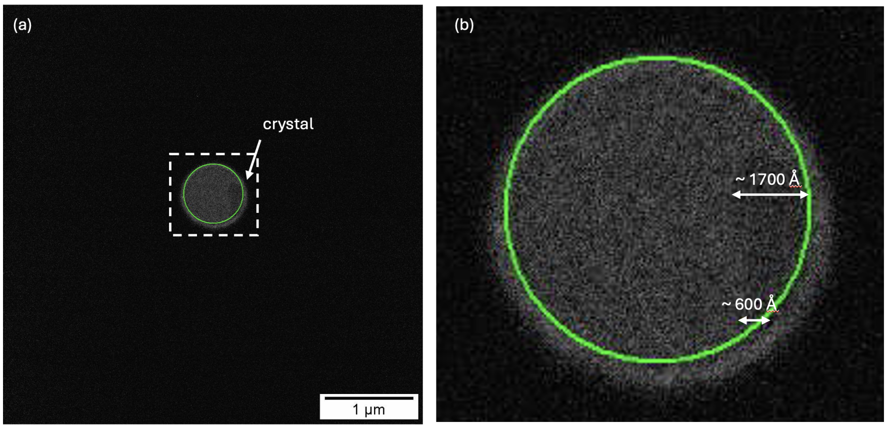

Fig. S9 Images of quartz crystals to provide rough estimate of dimension. (a) Image of the quartz crystal from which the experimental 3D-ED dataset used in the maintext is extracted with a selected area aperture in place (b) Zoom in of region shown in (a). Demonstrating the approximate size of the quartz crystal. Adopted from the dataset reported in [4, 5]. Estimating crystal size from this image is a qualitative analysis at best

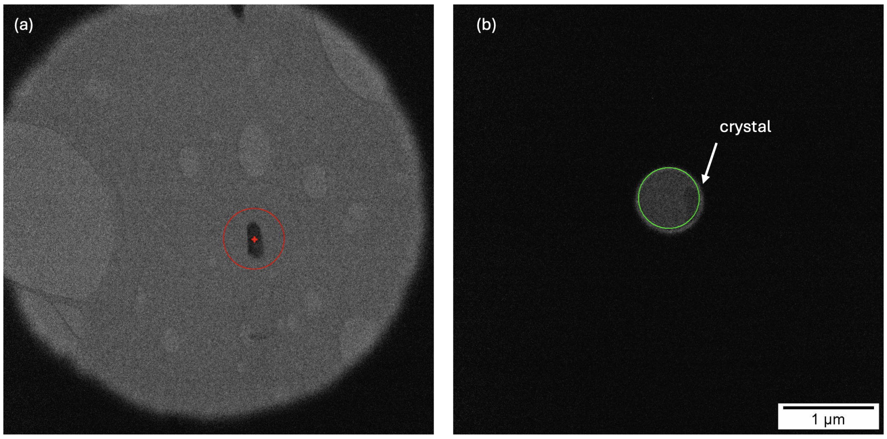

Fig. S10 Images of quartz crystal to demonstrate impact of selected area aperture on region of interest. (a) Quartz crystal from which the 3D-ED data utilised in the main text is extracted. (b) Same regions as (a) with a selected area aperture inserted. Data presented in this figure adopted from [4, 5]

References

[1] Suresh, A., Yörük, E., Cabaj, M.K., Brázda, P., Výborný, K., Sedláček, O., Müller, C., Chintakindi, H., Eigner, V., Palatinus, L.: Ionisation of atoms determined

by kappa refinement against 3D electron diffraction data. Nature Communications **15**(1), 9066 (2024) https://doi.org/10.1038/s41467-024-53448-2 . Publisher: Nature Publishing Group. Accessed 2025-05-01

[2] Kingma, D.P., Ba, J.: Adam: A method for stochastic optimization. In: Bengio, Y., LeCun, Y. (eds.) 3rd International Conference on Learning Representations, ICLR 2015, San Diego, CA, USA, May 7-9, 2015, Conference Track Proceedings (2015). http://arxiv.org/abs/1412.6980

[3] Liu, D.C., Nocedal, J.: On the limited memory BFGS method for large scale optimization. Math. Program. **45**(1-3), 503–528 (1989) https://doi.org/10.1007/BF01589116

[4] Klar, P.B., Krysiak, Y., Xu, H., Steciuk, G., Cho, J., Zou, X., Palatinus, L.: "Chirality and accurate structure models by exploiting dynamical effects in continuous-rotation 3D ED data". Raw data and JANA refinement files. Zenodo (2021). https://doi.org/10.5281/zenodo.7185657 . https://zenodo.org/records/7185657 Accessed 2026-03-07

[5] Klar, P.B., Krysiak, Y., Xu, H., Steciuk, G., Cho, J., Zou, X., Palatinus, L.: Accurate structure models and absolute configuration determination using dynamical effects in continuous-rotation 3D electron diffraction data. Nature Chemistry **15**(6), 848–855 (2023) https://doi.org/10.1038/s41557-023-01186-1 . Number: 6 Publisher: Nature Publishing Group. Accessed 2024-01-10
