# Supplementary material for: Hybrid physics-machine learning models for quantitative electron diffraction refinements
Source: Nat Commun. 2026 Apr 11;17:5056. doi: 10.1038/s41467-026-71673-9 (PMC13243467; doi:10.1038/s41467-026-71673-9)
Supplement: Supplementary file 2 — Description of Additional Supplementary Files [file 41467_2026_71673_MOESM2_ESM.pdf]

Description of Additional Supplementary Files

File Name: Supplementary Video 1

Description: Quartz crystal rotating during data acquisition
