# Supplementary material for: Hybrid physics-machine learning models for quantitative electron diffraction refinements
Source: Nat Commun. 2026 Apr 11;17:5056. doi: 10.1038/s41467-026-71673-9 (PMC13243467; doi:10.1038/s41467-026-71673-9)
Supplement: Supplementary file 4 — Transparent Peer Review file [file 41467_2026_71673_MOESM4_ESM.pdf]

nature portfolio

Peer Review File

Hybrid Physics-Machine Learning Models for Quantitative Electron Diffraction Refinements

Corresponding Author: Dr Tiarnan Doherty

This file contains all reviewer reports in order by version, followed by all author rebuttals in order by version.

Version 0:

Reviewer comments:

Reviewer #1

(Remarks to the Author)
“Hybrid Physics-Machine Learning Models for Quantitative Electron Diffraction Refinements” by Malik et al. describes a procedure for dynamical refinement of 3D ED data using machine learning techniques, exemplified by three test structures. The application of ML presented in the manuscript is unexpected, and, in my opinion, would generally require many more training examples to be justified. Nevertheless, the idea is interesting and definitely deserves publication.

Some minor remarks:
When citing the nanometer-scale crystals on page 3, top lines, a recent paper on nanocrystals can be mentioned: https://pubs.acs.org/doi/full/10.1021/acsnano.5c01764

Page 4, section 2.1: When reading the manuscript for the first time, I was missing some information here, which was provided later, but should be included in this section. Firstly, it should be stated that the data are of a continuous rotation type. Secondly, that reduced data – hkll – are matched.

Page 6, noise models: These may become inappropriate in the future, but at the moment it is sufficient that the assumptions are well documented.

Page 7, first paragraph: I was not familiar with the Frobenius norm and had to look it up. Perhaps this deserves a brief section in the ESI.

Page 7, second paragraph: “MicroED data”—this term appears suddenly here. Initially, you used “3D ED"; now "MicroED"—could you be consistent? "3D ED" seems to be a more general and appropriate term. It might be worth mentioning both at least at the beginning.

Page 8, section 8: It was not clear to me whether you refine on a list of reflections or on a semi-synthetic pattern—I already mentioned this above. Please clarify this earlier.

Page 9, second paragraph: “The first demonstration of an alternative code to Jana”—I have heard of multislice refinements of dynamical diffraction, though I am not sure if anything has been published. Anyway, it can be mentioned here.

A general question: In my understanding, the model of dynamical scattering has to be trained for each structure before refinement. Could the authors consider transferable models? Perhaps a short section in the outlook would be appropriate.

(Remarks on code availability)

Reviewer #2

(Remarks to the Author)
This manuscript presents a proof-of-principle application of a differentiable hybrid physics model for the aim of improving the dynamical refinement of electron crystallography structures. I am generally impressed by the originality of the over-arching

aim and application of this methodology and I agree with the authors that this is a timely and important step for work in this field.

My main question for the authors is a deeper discussion of the impact of their work on the refinement procedure. For the experimental data section the thicknessNN approach makes a small difference to the final R-factor (in the case of paracetamol it improves one metric and makes another worse). If this approach is to be adopted widely and replicated for other systems, a more concrete measure of the improvement it offers would be useful. If we accept that the thicnes profiles are more accurate (and in two cases they look very plausible), however in only one case does the returned thickness profile have much coincidence with the 'single value' thickness used in the convetional dynamical refinement. I guess my question is a wider one of why there is such a large discrepancy in the returned thicknesses? I don't know if the authors have a plot of the dynamical refinement R-factors as a function of thickness for single thickness value but could it be that there is a wide shallow minimum that encompasses the full range of results shown?

This extends through to the thickness profile of the quartz crystal. The authors comment that the crystal is shown to move in the beam but in the video clip it appears to be fairly uniformly illuminated at all times and appears to be a fairly simple convex shape, while the thickness profile suggests something more complex with protrusions or a more concave surface. I'm not sure that the discussion presented fully addreses the situation. My other query on this video is that there is another smaller object in the field of view and there is a region of the tilting where a neighbouring crystal is illuminated. How does this affect the results? I'm not expecting there to be perfect alignment of the lattices but the addition of any scattering can affect the local background and may lead to overlap of reflections that would perturb any integration of intensities. If the aim is to describe the sample as faithfully as possible I think these considerations/limitations should be addressed explicitly.

Extending this back to the synthetic data, the fact that the thicknessNN recovers the thickness profile so accurately suggests that in truth the limitation to refinement quality (or at least to how low you can make the R-factors, which may or may not be the same thing) is another quality of the crystal and not just the thickness. I think that taking care to address the question of thickness allows the authors to comment more authoritatively on the other sources of error in the modelling of the crystal or the calcuation of the diffraction.

This does not take away from the technical achievement of the improved phyics modelling but ultimately for a paper that addresses both the methodolgy and a practical application such discussion is essential to ensure the correct impact of the work.

(Remarks on code availability)
The code is not currently available. I trust the authors statement that it is being developed for future release.

Version 1:

Reviewer comments:

Reviewer #1

(Remarks to the Author)

(Remarks on code availability)

Reviewer #2

(Remarks to the Author)
Thank you for considering the original review comments. I am confident my queries have been addressed and I'm happy to accept the revised manuscript.

(Remarks on code availability)
There seems to be sufficient information to run the code, I was able to get the demo to run. I haven't yet reproduced all of the findings of the paper.

Open Access This Peer Review File is licensed under a Creative Commons Attribution 4.0 International License, which permits use, sharing, adaptation, distribution and reproduction in any medium or format, as long as you give appropriate credit to the original author(s) and the source, provide a link to the Creative Commons license, and indicate if changes were made.
In cases where reviewers are anonymous, credit should be given to 'Anonymous Referee' and the source.
The images or other third party material in this Peer Review File are included in the article's Creative Commons license, unless indicated otherwise in a credit line to the material. If material is not included in the article's Creative Commons license and your intended use is not permitted by statutory regulation or exceeds the permitted use, you will need to obtain permission directly from the copyright holder.
To view a copy of this license, visit https://creativecommons.org/licenses/by/4.0/

We are pleased to submit a revised version of our manuscript, “Hybrid Physics–Machine Learning Models for Quantitative Electron Diffraction Refinements.”

We thank the reviewers for their thoughtful evaluation. We were greatly encouraged by their feedback describing the work as “interesting and definitely deserves publication,” and by the recognition of its “originality” and position as “a timely and important step” for the field. We believe their comments have substantially improved the clarity and quality of the manuscript.

In addition to addressing the reviewers’ specific requests, we have taken the opportunity to make further improvements to the manuscript including:

1. **Clarification of the Modeling Approach vs. Previous Methods:** Following a communication regarding our preprint, specifically concerning the differences between our approach and previous methods, we have significantly expanded the exposition of the modeling framework and added a new figure (Fig. 3). We believe delving into these distinctions provides necessary context and increases the utility of the manuscript for the wider community, while also addressing a number of reviewer 1’s comments.
2. **Correction to Synthetic Data Weighting:** During our own internal validation, we identified that the wR weighting used in the original synthetic data experiments was slightly incorrect. To ensure absolute rigor, we have re-run the complete suite of synthetic experiments with the corrected weighting. This correction has **not** changed our scientific conclusions, but the relevant figures and results tables have been updated to reflect the most accurate data.

A detailed, point-by-point response to all reviewer comments is provided below.

REVIEWER COMMENTS

Reviewer #1 (Remarks to the Author):

“Hybrid Physics-Machine Learning Models for Quantitative Electron Diffraction Refinements” by Malik et al. describes a procedure for dynamical refinement of 3D ED data using machine learning techniques, exemplified by three test structures. The application of ML presented in the manuscript is unexpected, and, in my opinion, would generally require many more training examples to be justified. Nevertheless, the idea is interesting and definitely deserves publication.

We thank the reviewer for the positive assessment of the manuscript deeming it worthy of publication. The concern around training examples we believe may stem from a misunderstanding of the role of the machine-learning components in our framework. Our method does not aim to train a model that generalizes across

experiments; instead, we fit a lightweight neural network specific to each individual dataset in order to efficiently parameterize the forward model. In this sense, the network plays the role of a differentiable surrogate for experimental parameters such as thickness, which in previous studies have been manually tuned. The Bloch wave dynamical scattering model itself is not learned and is simply a restatement of the Bloch wave equations, as in existing refinement packages (Figure 2). We have clarified this distinction in the introduction and Section 2.1 and 2.2 of the revised manuscript and added a new Figure (Figure 3).

We show that our method works effectively on three structurally and compositionally distinct datasets, which include organic, inorganic, and heavy-element systems, with both isotropic and anisotropic thermal displacement parameters, to showcase both the robustness and the general applicability of the approach. These datasets collectively span a very large proportion of the types of materials systems that would be commonly encountered in typical 3D-ED experiments. To improve the clarity of the paper, we have substantially expanded the background explanation of the refinement procedure and the role of the synthetic data used during optimization in Section 2.2.

Some minor remarks:

When citing the nanometer-scale crystals on page 3, top lines, a recent paper on nanocrystals can be mentioned:
https://pubs.acs.org/doi/full/10.1021/acsnano.5c01764
We thank the reviewer for pointing out this relevant citation, we have added it to the paper.

Page 4, section 2.1: When reading the manuscript for the first time, I was missing some information here, which was provided later, but should be included in this section. Firstly, it should be stated that the data are of a continuous rotation type.
.
We have now clarified this in section 1 with the line:
All datasets contained in this paper are continuous rotation type datasets, with the exception of the Paracetamol experimental dataset presented in section 2.3, which is a precession assisted rotation dataset.

Secondly, that reduced data – hkl – are matched.

We have now clarified this in section 2.1. We removed the original sentence “*The refinement proceeds against integrated intensities and the data is pre-processed according to previously reported virtual frame methods developed by [DOI: 10.1107/S2052520619007534, DOI: 10.1107/S010876731204946X, DOI: 10.1107/S2053273315001266 and DOI: 10.1038/s41557-023-01186-1].*”

and replaced it with:

"*The refinement is carried out against experimental integrated intensities, obtained after the full reduction of the raw diffraction patterns. This involves indexing and extracting per-hkl intensities with uncertainties informed by detector gain and noise characteristics, and the variance obtained from the background pixels surrounding each Bragg disc [DOI: 10.1107/S2052520619007534]. The data are then organised into virtual frames by segmenting the tilt series into angular ranges, and each hkl is allocated to the appropriate frame to allow direct matching of simulated and experimental hkl, with refinements carried out on a frame-by-frame basis, as described in [DOI: 10.1107/S2052520619007534], DOI: 10.1107/S010876731204946X, DOI: 10.1107/S2053273315001266, DOI: 10.1038/s41557-023-01186-1}.* "

Page 6, noise models: These may become inappropriate in the future, but at the moment it is sufficient that the assumptions are well documented.

Thank you for this comment. We believe there may have been some confusion regarding our use of noise models in the paper and how they could have impacted results on experimental data. To clarify: our noise model is used only in the synthetic *benchmarks* of our methodology to add Poisson counting noise (such as what would be encountered in a direct electron detector) and to avoid an artificially easy task. It plays no role in the experimental refinements. For *experimental* refinement we simulate diffracted intensities and use these intensities to refine our model against the experimentally observed integrated intensities. No noise is added to the simulated intensities as is standard practice in other crystallographic refinement workflows, including those previously reported for the refinement of 3D-ED data. Noise in the experimental intensities is treated according to protocols established in *DOI: 10.1107/S2052520619007534*.

Additionally, we note the limitations of assuming Poisson noise as the only source of noise in our benchmark synthetic data experiments at the beginning of section 2.3 and how the true test is a refinement on real experimental data, which is subject to additional noise sources. We firmly agree with the reviewer that advanced noise models applied to the dynamical refinement of 3D-ED in the future are an exciting area of research and one that a differentiable diffraction framework uniquely enables and have highlighted this in the updated Discussion section.

Page 7, first paragraph: I was not familiar with the Frobenius norm and had to look it up. Perhaps this deserves a brief section in the ESI.

We have added a subsection to the Methods section defining the Frobenius norm and its use in this work.

Page 7, second paragraph: “MicroED data”—this term appears suddenly here. Initially, you used “3D ED"; now "MicroED"—could you be consistent? "3D ED" seems to be a more general and appropriate term. It might be worth mentioning both at least at the beginning.

Thank you for bringing this to our attention, we have edited the manuscript to use 3D-ED throughout the text.

Page 8, section 8: It was not clear to me whether you refine on a list of reflections or on a semi-synthetic pattern—I already mentioned this above. Please clarify this earlier.
This comment is closely related to the earlier point about matching simulated and experimental intensities. As noted in our response to that comment, and as clarified in Section 2.1, the manuscript now states explicitly that refinements are performed on reduced lists of experimentally-obtained integrated reflections, with synthetic data used only in clearly identified benchmark sections.

Page 9, second paragraph: “The first demonstration of an alternative code to Jana”—I have heard of multislice refinements of dynamical diffraction, though I am not sure if anything has been published. Anyway, it can be mentioned here.

To the best of our knowledge, no other codebase has been publicly demonstrated for the dynamical refinement of 3D electron diffraction (3D-ED) data aside from Jana. Performing such refinements requires the simulation of fully integrated intensities over small crystal tilts. While various codebases have been developed to refine non-integrated dynamical diffraction patterns collected at major crystallographic zone axes, these approaches are fundamentally different from the integrated-intensity refinement methodology implemented in Jana and applied here. There may of course be unpublished results (or results that we have missed) that utilise multislice to perform refinement of 3D-ED data but at this time we are not aware of them. To clarify this, we have added the following text and citations to the paper

Other dynamical refinement codebases [31, 42–44] in the past have focused on the refinement of non-integrated intensities extracted from individual diffraction patterns (often convergent beam electron diffraction) collected near major crystallographic zone
axes, which is a fundamentally different problem to the one presented here.

A general question: In my understanding, the model of dynamical scattering has to be trained for each structure before refinement. Could the authors consider transferable models? Perhaps a short section in the outlook would be appropriate.

This point relates closely to the reviewer’s first comment and we believe stems from a misunderstanding of the role and motivation of our method. In our approach, the

dynamical scattering model itself is not learned and does not require training - we have explicitly implemented the Bloch wave equations as a deterministic forward model, as in conventional dynamical refinements.

The learned component is used solely to parameterize experimental, *sample-specific* effects such as orientation-dependent thickness variations that are difficult to model analytically but strongly influence the observed intensities. The refinement therefore proceeds analogously to standard refinements, except that experimental parameters are jointly optimised with the structural parameters. Transferability between samples is therefore not a consideration in our approach, just like it is not a consideration in traditional crystallographic refinements.

We have clarified the role and motivation of the (trainable) thickness neural network and the (fixed, deterministic) differentiable Bloch wave simulation in the Introduction and Section 2.1 and 2.2. While transferable learned components could be explored in future work, this is separate to the present goal of quantitative, physically grounded refinements in this work.

Reviewer #2 (Remarks to the Author):
This manuscript presents a proof-of-principle application of a differentiable hybrid physics model for the aim of improving the dynamical refinement of electron crystallography structures. I am generally impressed by the originality of the over-arching aim and application of this methodology and I agree with the authors that this is a timely and important step for work in this field.

My main question for the authors is a deeper discussion of the impact of their work on the refinement procedure. For the experimental data section the thicknessNN approach makes a small difference to the final R-factor (in the case of paracetamol it improves one metric and makes another worse). If this approach is to be adopted widely and replicated for other systems, a more concrete measure of the improvement it offers would be useful. If we accept that the thicnes profiles are more accurate (and in two cases they look very plausible), however in only one case does the returned thickness profile have much coincidence with the 'single value' thickness used in the convetional dynamical refinement. I guess my question is a wider one of why there is such a large discrepancy in the returned thicknesses?
I don't know if the authors have a plot of the dynamical refinement R-factors as a function of thickness for single thickness value but could it be that there is a wide shallow minimum that encompasses the full range of results shown?

We thank the reviewer for highlighting the discrepancy between the recovered thickness profile and the single thickness used in the conventional dynamical refinements. The first thing to highlight is that the single thickness value is not one that we produced with our refinement software. Rather, it is the thickness extracted

from reported dynamical refinements in the literature. Taking the example of quartz, this corresponds to the following publication by Klar et al. [DOI: 10.1038/s41557-023-01186-1]. An explanation for the discrepancy between their results and ours could be that the quartz dataset is very ‘kinematical like’ in nature and as a result, diffracted intensities do not exhibit a strong dependence on thickness, which, as the reviewer notes, could lead to a wide shallow minimum that encompasses the range of results shown. To interrogate this further, for a range of single thicknesses, we plotted the R-factor extracted from our own simulator averaged across all orientations in the dataset. As can be seen in the newly added Supplementary Figure 8, a clear minimum can be seen at ~870Å. This value compares very closely to the mean value of the thickness profile extracted with our ThicknessNN. As to why the recovered single thickness varies so greatly when compared to the one reported in the literature, it is challenging to say. Interestingly however, if we interrogate an image of the crystal from which the Quartz 3D-ED dataset is extracted (https://zenodo.org/records/7185657) and attempt to measure the width of the crystal (newly added Supplementary Figure 9) then we observe that at its widest point it is ~1700Å wide and at its narrowest it is ~600Å. While this is at best a qualitative observation, it does suggest that our larger recovered thickness value may be a closer approximation to the true crystal thickness. To clarify this, we have added Supplementary Note 4 in addition to Supplementary Figures 8 and 9. In addition, we have added the following to the main text.

The discrepancy between these thickness values is difficult to explain. Notably though, if we turn off the ThicknessNN and use a single thickness value in our simulator, we observe a minimum in wRall at ~87.2 nm (Supplementary Figure S8) which compares very closely with the mean thickness recovered by the ThicknessNN. Additionally, interrogating images of the crystal from which the 3D-ED dataset is recorded - though a qualitative analysis at best - suggests that the true mean crystal thickness is likely closer to ~85 nm than 44 nm (See Supplementary Figure S9 and Supplementary Note 4).

The reviewer also mentions that at least two of the three recovered thickness profiles look plausible. We suspect that the third profile which perhaps looks less plausible to the reviewer, likely belongs to paracetamol. This puzzled us too. The dataset used for paracetamol originates from the online tutorial at the following link (http://pets.fzu.cz/download/tutorials/Paracetamol.pdf). To our knowledge, the dataset is not directly linked to a publication. However, it is possible that it is related to the dataset utilised in the following publication from the same authors [DOI: 10.1126/science.aak9652]. In that publication, Fig 1A clearly shows movement of the electron beam across the sample during data acquisition, which could have led to the thickness profile we extract from paracetamol in our work. To clarify this, we have added the following to the main text

…or alternatively, could be explained by the electron beam intentionally being moved across the sample during acquisition to mitigate beam damage as previously reported [DOI: 10.1126/science.aak9652].

More broadly, we emphasize that the aim of this manuscript is not to claim universally improved refinement accuracy over established dynamical approaches *at this stage*, but rather to demonstrate that hybrid physics–machine learning models can be successfully embedded within crystallographic refinement workflows for electron microscopy and offer substantial practical benefits. In the specific case of the ThicknessNN, the principal advantage is the increased flexibility it affords over traditional rigid geometric thickness models, allowing experimentally realistic heterogeneity to be learned directly from the data, simultaneously to atomic position refinement, without requiring prior assumptions about sample shape. We have substantially revised Sections 1 and 2 of the manuscript to clarify this motivation and to better articulate how differentiable hybrid models enable such flexibility while remaining compatible with standard refinement procedures.

While we anticipate that the ThicknessNN approach will ultimately enable more accurate thickness modeling than conventional methods, rigorous evaluation of this claim will require quantitative refinement on specimens with well-defined geometries, for example FIB-milled samples of known thickness - which we believe is beyond the scope of this current work. Looking forward, we do anticipate that extending similar hybrid strategies to other sources of forward model inadequacy will enable progressively more accurate refinements and facilitate the reliable extraction of subtle physical features from electron diffraction data, such as charge-density deformation due to bonding. We have revised the discussion portion of the paper to include some of these thoughts.

This extends through to the thickness profile of the quartz crystal. The authors comment that the crystal is shown to move in the beam but in the video clip it appears to be fairly uniformly illuminated at all times and appears to be a fairly simple convex shape, while the thickness profile suggests something more complex with protrusions or a more concave surface. I'm not sure that the discussion presented fully addreses the situation. My other query on this video is that there is another smaller object in the field of view and there is a region of the tilting where a neighbouring crystal is illuminated. How does this affect the results? I'm not expecting there to be perfect alignment of the lattices but the addition of any scattering can affect the local background and may lead to overlap of reflections that would perturb any integration of intensities. If the aim is to describe the sample as faithfully as possible I think these considerations/limitations should be addressed explicitly.

We thank the reviewer for pointing this out. The inclusion of the supplementary video was confusing without providing further context and figures. Indeed we would have

raised the same comments as the reviewer had we been in their position. We apologise for this oversight. The supplementary video was reconstructed from images extracted from the quartz folder at the following link (https://zenodo.org/records/7185657) which contains data associated with a publication by Klar et al. [10.1038/s41557-023-01186-1] and is not our own data. The image frames used to create the supplementary video are from a 'crystal tracking' experiment described in the Methods section of the same paper [10.1038/s41557-023-01186-1] in which 21 TEM images are recorded to track the quartz crystal across the experimental tilt range from -50° to +50°. From these images, the idealized movement of the crystal during acquisition is interpolated. For data acquisition, the authors appear to utilise a selected area aperture - an image of the probe after aperture insertion has been now added in Supplementary Fig 9 and 10 - which would serve to exclude any scattering from neighbouring crystals. The authors also state that during acquisition, the beam position is adapted to the expected crystal position (without switching to image mode) such that the crystal is illuminated throughout acquisition. As is visible in the image of the probe (panel b supplementary figures 9 and 10), the illuminated area is not much larger than the crystal of interest and it is *possible* that part of the crystal may have moved out of the area of illumination at some point during data acquisition. In fact this appears to be the case in panel b Fig S10, though the contrast makes it difficult to say for certain. This, combined with what we interpret as a concave rather than a convex specimen geometry on the bottom of the crystal, could lead to the relatively complex thickness profile recovered from our experiments. As we did not acquire the data ourselves, we are hesitant to speculate too much on how the recovered thickness profile relates to specimen geometry. As mentioned in the response preceding this one, obtaining datasets with known specimen geometries in the future will allow us to determine, quantitatively, how much information about the specimen geometry can practically be recovered from experimental diffraction intensities.The main aim in this work is to demonstrate that hybrid-physics machine learning can be utilised in the refinement of 3D-ED data and can achieve comparable performance to traditional methods but with increased flexibility. We have added Supplementary Note 5 to the paper in addition to Supplementary Figures 9 and 10. We have also modified the main text to read

The recovered thickness distribution (Figure. 6) is plausible given the complex geometry of the quartz crystal and possible specimen movement during data acquisition (See Supplementary Video 1 and Supplementary Note 5). However, we note that it is difficult to derive accurate 3D specimen geometries from experimental electron microscopy images without a full tomographic analysis.

Extending this back to the synthetic data, the fact that the thicknessNN recovers the thickness profile so accurately suggests that in truth the limitation to refinement quality (or at least to how low you can make the R-factors, which may or may not be the same thing) is another quality of the crystal and not just the thickness. I think that

taking care to address the question of thickness allows the authors to comment more authoritatively on the other sources of error in the modelling of the crystal or the calcuation of the diffraction.

We agree with the reviewer's assessment. While we are currently hesitant to assign a quantitative accuracy to the experimentally refined thickness models for the reasons discussed above, the near-perfect recovery of ground-truth thickness in the synthetic data strongly suggests that, once thickness variations are properly accounted for, other factors become the dominant limitations on refinement quality and achievable *R*-factors. We anticipate that the accuracy of the thickness refinement itself can be rigorously evaluated in the near future using experimental specimens of known thickness and geometry.

Beyond thickness variations, additional physical effects and modeling assumptions, including inelastic scattering, detector noise, crystal defects, beam damage, and the independent atom approximation, are expected to influence both the accuracy of the recovered structure and the minimum attainable *R*-factor. We have therefore substantially revised the Discussion to emphasize these remaining sources of error and to outline how a hybrid physics–machine learning framework provides a natural and extensible platform for systematically incorporating and evaluating their impact in future refinements.

This does not take away from the technical achievement of the improved phyics modelling but ultimately for a paper that addresses both the methodolgy and a practical application such discussion is essential to ensure the correct impact of the work.

We agree and thank the reviewer again for their comments. We believe that they have strengthened the paper substantially.

Reviewer #2 (Remarks on code availability):

The code is not currently available. I trust the authors statement that it is being developed for future release.

We apologise for this, we had submitted a version of the codebase alongside our original submission capable of reproducing the work presented here for the purpose of review. We have now decided to instead place a version of the codebase on Zenodo at the following doi: 10.5281/zenodo.18281349 to make it available with the paper release. We have modified the code availability statement in the paper. We are still actively working on a mature, stable and easier to use version of the codebase with software engineering for release in the near future on github.
